# Di-μ-chlorido-bis­[(2,2′:6′,2′′-terpyridine-κ^3^ *N*,*N*′,*N*′′)copper(II)] bis­(tri­fluoro­methane­sulfonate)

**DOI:** 10.1107/S2414314621010968

**Published:** 2021-10-21

**Authors:** Rafael A. Adrian, Jose J. Duarte, Hadi D. Arman

**Affiliations:** a Department of Chemistry and Biochemistry, University of the Incarnate Word, San Antonio TX 78209, USA; b Department of Chemistry, The University of Texas at San Antonio, San Antonio TX 78249, USA; Goethe-Universität Frankfurt, Germany

**Keywords:** crystal structure, terpyridine, copper, tri­fluoro­methane­sulfonate salt, bridging chloride, *π*–*π* stacking

## Abstract

In the centrosymmetric title complex, [Cu_2_Cl_2_(C_15_H_11_N_3_)_2_](CF_3_O_3_S)_2_, the Cu^II^ metal center is fivefold coordinated by two chloride ions and three nitro­gen atoms of the terpyridine ligand in a distorted square-pyramidal geometry; two tri­fluoro­methane­sulfonate ions complete the outer coordination sphere. *π–π* stacking inter­actions between the pyridyl rings in adjacent mol­ecules contribute to the alignment of the complexes in columns along the *a*-axis. This structure represents the first example of a binuclear dication of formula [Cu(terpy)_2_Cl_2_]^2+^ with tri­fluoro­methane­sulfonate as counter-ions.

## Abstract 2

The crystal structure of the centrosymmetric complex [Cu(terpy)_2_Cl_2_](OTF)_2_ consists of a Cu^II^ metal center in a distorted square-pyramidal geometry with *π*–*π* stacking inter­actions contributing to the crystal packing.

## Graphical abstract

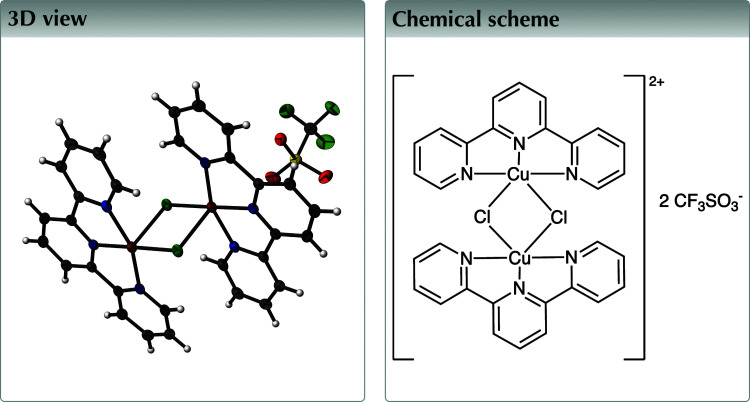

## Structure description

Terpyridines are some of the most studied nitro­gen-based tridentate ligands in coordin­ation chemistry, and their metal complexes have found application in catalysis (Wei *et al.*, 2019 ▸; Choroba *et al.*, 2019 ▸), supra­molecular chemistry (Wei *et al.*, 2019 ▸), and medicinal chemistry (Glišić *et al.*, 2018 ▸; Malarz *et al.*, 2021 ▸; Li *et al.*, 2020 ▸). Recently, copper(II) terpyridine complexes have received much attention due to their remarkable cytotoxicity and ability to inter­act with DNA (Karges *et al.*, 2021 ▸); herein, we report the synthesis and structure of the title copper(II) terpyridine complex.

**Figure 1:**
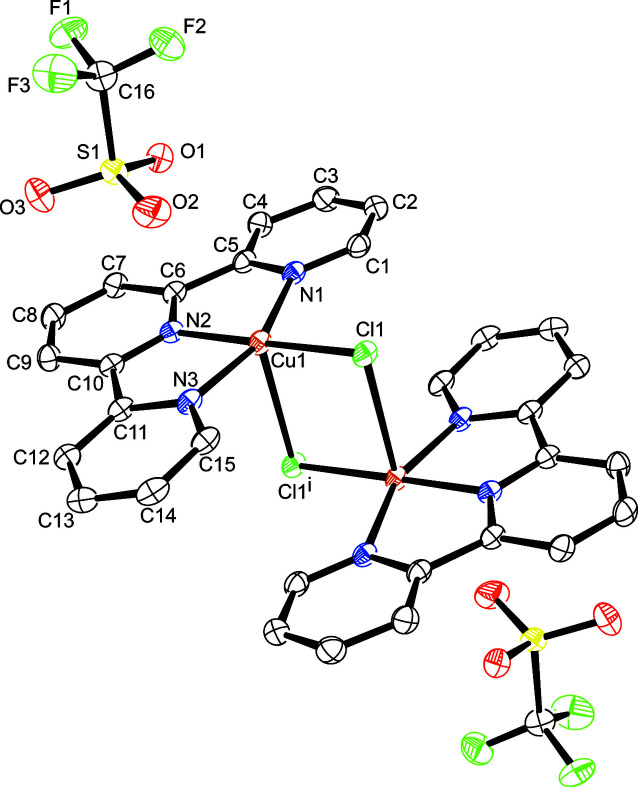
The mol­ecular structure of the title compound with displacement ellipsoids drawn at the 50% probability level; H atoms are omitted for clarity. Symmetry operator for generating equivalent atoms: (i) 1 − *x*, 1 − *y*, 1 − *z*.

The asymmetric unit of the title compound, depicted in Fig. 1 ▸, consists of half of a centrosymmetric dication [Cu(terpy)_2_Cl_2_]^2+^ and one tri­fluoro­methane­sulfonate ion completing the outer coordination sphere. The Cu—N, and Cu—Cl distances, as well as, the Cl—Cu—Cl, N—Cu—Cl and N—Cu—N angles are in good agreement with the reported values in similar copper(II) terpyridine complexes currently available in the CSD (version 5.42 with update September 2021; Rojo *et al.*, 1987 ▸; refcode FECJEC; Valdés-Martínez *et al.*, 2002; ▸ refcode HULZAP; Gasser *et al.*, 2004 ▸; refcode HULZAP01). All relevant bond lengths and angles involving the Cu atom are presented in Table 1 ▸.

In the crystal packing of the title compound, *π*–*π* stacking inter­actions between the N1 and N3 pyridyl ring of adjacent mol­ecules are observed, with a centroid-to-centroid (*Cg*⋯*Cg*) distance of 3.658 (1) Å and an offset distance of 1.723 Å. No other supra­molecuar inter­action is present in the crystal packing of the title compound.

## Synthesis and crystallization

The title compound was obtained as product of the reaction of 2,2′:6′,2′′-terpyridine (0.100 g, 0.429 mmol) with copper(II) chloride dihydrate (0.073 g, 0.429 mmol) in aceto­nitrile after the addition of silver tri­fluoro­methane­sulfonate (0.110 g, 0.429 mmol) and filtration using a 0.45 µm PTFE syringe filter. Crystals suitable for X-ray diffraction of the title compound were obtained by vapor diffusion of diethyl ether over the resulting aceto­nitrile solution at 278 K.

## Refinement

Crystal data, data collection and structure refinement details are summarized in Table 2 ▸. H atoms were located in a difference map and refined in idealized positions using a riding model with atomic displacement parameters of *U*_iso_(H) = 1.2*U*_eq_(C) and with a C—H distance of 0.95 Å.

## Supplementary Material

Crystal structure: contains datablock(s) I. DOI: 10.1107/S2414314621010968/bt4119sup1.cif

Structure factors: contains datablock(s) I. DOI: 10.1107/S2414314621010968/bt4119Isup2.hkl

Supporting information file. DOI: 10.1107/S2414314621010968/bt4119Isup3.mol

CCDC reference: 2116881

Additional supporting information:  crystallographic information; 3D view; checkCIF report

## Figures and Tables

**Figure 2:**
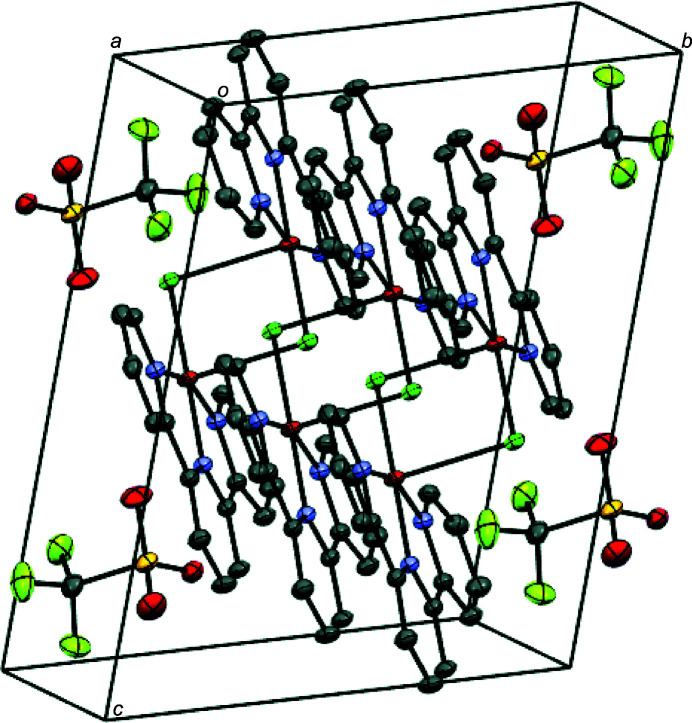
Perspective view of the packing structure of the title complex along the crystallographic *a*-axis; H atoms are omitted for clarity.

**Table 1:** Selected geometric parameters (Å, °)

Cu1—Cl1	2.2265 (5)	Cu1—N2	1.9420 (17)
Cu1—Cl1^i^	2.7660 (6)	Cu1—N1	2.0397 (18)
Cu1—N3	2.0278 (19)		
			
Cl1—Cu1—Cl1^i^	89.944 (18)	N2—Cu1—N3	80.39 (7)
N3—Cu1—Cl1^i^	90.30 (5)	N2—Cu1—N1	80.11 (7)
N3—Cu1—Cl1	99.82 (5)	N1—Cu1—Cl1	99.60 (5)
N3—Cu1—N1	159.58 (7)	N1—Cu1—Cl1^i^	95.97 (5)
N2—Cu1—Cl1^i^	90.83 (5)	Cu1—Cl1—Cu1^i^	90.056 (18)
N2—Cu1—Cl1	179.20 (5)		

Symmetry code: (i) 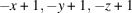 .

**Table 2:** Experimental details

Crystal data	
Chemical formula	[Cu_2_Cl_2_(C_15_H_11_N_3_)_2_](CF_3_O_3_S)_2_
*M*_r_	962.65
Crystal system, space group	Triclinic, *P* 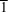
Temperature (K)	98
*a*, *b*, *c* (Å)	7.2767 (2), 9.8394 (2), 13.1746 (3)
α, β, γ (°)	106.667 (2), 91.226 (2), 105.453 (2)
*V* (Å^3^)	866.08 (4)
*Z*	1
Radiation type	Mo *K*α
μ (mm^−1^)	1.59
Crystal size (mm)	0.47 × 0.17 × 0.1

Data collection	
Diffractometer	XtaLAB AFC12 (RCD3): Kappa single
Absorption correction	Multi-scan (*CrysAlis PRO*; Rigaku OD, 2019 ▸)
*T*_min_, *T*_max_	0.741, 1.000
No. of measured, independent and observed [*I* > 2σ(*I*)] reflections	33748, 3982, 3889
*R*_int_	0.047
(sin θ/λ)_max_ (Å^−1^)	0.650

Refinement	
*R*[*F* ^2^ > 2σ(*F* ^2^)], *wR*(*F* ^2^), *S*	0.036, 0.094, 1.08
No. of reflections	3982
No. of parameters	253
H-atom treatment	H-atom parameters constrained
Δρ_max_, Δρ_min_ (e Å^−3^)	0.55, −0.41

Computer programs: *CrysAlis PRO* (Rigaku OD, 2019 ▸), *SHELXT* (Sheldrick, 2015*a* ▸), *SHELXL2018/3* (Sheldrick, 2015*b* ▸), and *OLEX2* (Dolomanov *et al.*, 2009 ▸).

## full crystallographic data

### Crystal data

**Table:**

[Cu_2_Cl_2_(C_15_H_11_N_3_)_2_](CF_3_O_3_S)_2_	*Z* = 1
*M**_r_* = 962.65	*F*(000) = 482
Triclinic, *P*1	*D*_x_ = 1.846 Mg m^−^^3^
*a* = 7.2767 (2) Å	Mo *K*α radiation, λ = 0.71073 Å
*b* = 9.8394 (2) Å	Cell parameters from 14071 reflections
*c* = 13.1746 (3) Å	θ = 3.1–29.5°
α = 106.667 (2)°	µ = 1.59 mm^−^^1^
β = 91.226 (2)°	*T* = 98 K
γ = 105.453 (2)°	Block, clear bluish green
*V* = 866.08 (4) Å^3^	0.47 × 0.17 × 0.1 mm

### Data collection

**Table:**

XtaLAB AFC12 (RCD3): Kappa single diffractometer	3889 reflections with *I* > 2σ(*I*)
Radiation source: Rotating-anode X-ray tube, Rigaku (Mo) X-ray Source	*R*_int_ = 0.047
ω scans	θ_max_ = 27.5°, θ_min_ = 2.3°
Absorption correction: multi-scan (CrysAlisPro; Rigaku OD, 2019)	*h* = −9→9
*T*_min_ = 0.741, *T*_max_ = 1.000	*k* = −12→12
33748 measured reflections	*l* = −16→17
3982 independent reflections	

### Refinement

**Table:**

Refinement on *F*^2^	Primary atom site location: dual
Least-squares matrix: full	Hydrogen site location: inferred from neighbouring sites
*R*[*F*^2^ > 2σ(*F*^2^)] = 0.036	H-atom parameters constrained
*wR*(*F*^2^) = 0.094	*w* = 1/[σ^2^(*F*_o_^2^) + (0.0529*P*)^2^ + 0.6752*P*] where *P* = (*F*_o_^2^ + 2*F*_c_^2^)/3
*S* = 1.08	(Δ/σ)_max_ = 0.002
3982 reflections	Δρ_max_ = 0.55 e Å^−^^3^
253 parameters	Δρ_min_ = −0.41 e Å^−^^3^
0 restraints	

### Special details

**Table:**

Geometry. All esds (except the esd in the dihedral angle between two l.s. planes) are estimated using the full covariance matrix. The cell esds are taken into account individually in the estimation of esds in distances, angles and torsion angles; correlations between esds in cell parameters are only used when they are defined by crystal symmetry. An approximate (isotropic) treatment of cell esds is used for estimating esds involving l.s. planes.
Refinement. H atoms were located in a difference map and refined in idealized positions using a riding model with atomic displacement parameters of *U*_iso_(H) = 1.2*U*_eq_(C) and with a C—H distance of 0.95 Å.

### Fractional atomic coordinates and isotropic or equivalent isotropic displacement parameters (Å^2^)

**Table:**

	*x*	*y*	*z*	*U*_iso_*/*U*_eq_	
Cu1	0.49265 (4)	0.41355 (3)	0.60172 (2)	0.01772 (10)	
Cl1	0.62384 (8)	0.36614 (6)	0.44902 (4)	0.02026 (13)	
S1	0.50126 (8)	0.15121 (6)	0.79698 (4)	0.02142 (13)	
F2	0.7785 (2)	0.02432 (18)	0.78751 (14)	0.0396 (4)	
F1	0.6642 (2)	0.06787 (19)	0.93864 (12)	0.0395 (4)	
F3	0.5041 (2)	−0.11260 (17)	0.80642 (16)	0.0478 (4)	
O1	0.6469 (2)	0.29199 (17)	0.83604 (12)	0.0239 (3)	
O3	0.3333 (2)	0.1385 (2)	0.85354 (15)	0.0336 (4)	
O2	0.4661 (3)	0.0941 (2)	0.68265 (13)	0.0360 (4)	
N3	0.2255 (3)	0.27262 (19)	0.55326 (14)	0.0188 (4)	
N2	0.3802 (3)	0.45334 (19)	0.73563 (14)	0.0168 (3)	
N1	0.7290 (3)	0.55151 (19)	0.70135 (14)	0.0184 (4)	
C11	0.1030 (3)	0.2851 (2)	0.62992 (16)	0.0192 (4)	
C5	0.6901 (3)	0.6111 (2)	0.80261 (16)	0.0189 (4)	
C10	0.1958 (3)	0.3840 (2)	0.73629 (16)	0.0186 (4)	
C6	0.4902 (3)	0.5499 (2)	0.82254 (16)	0.0179 (4)	
C9	0.1110 (3)	0.4087 (2)	0.83124 (17)	0.0220 (4)	
H9	−0.019262	0.358669	0.833457	0.026*	
C15	0.1555 (3)	0.1851 (2)	0.45439 (17)	0.0220 (4)	
H15	0.240897	0.174252	0.401071	0.026*	
C14	−0.0386 (3)	0.1094 (2)	0.42706 (18)	0.0238 (5)	
H14	−0.084305	0.047636	0.356325	0.029*	
C12	−0.0910 (3)	0.2144 (2)	0.60811 (17)	0.0212 (4)	
H12	−0.173957	0.225959	0.662670	0.025*	
C13	−0.1631 (3)	0.1253 (2)	0.50390 (18)	0.0231 (4)	
H13	−0.296333	0.076425	0.486484	0.028*	
C7	0.4138 (3)	0.5820 (2)	0.91921 (16)	0.0208 (4)	
H7	0.489630	0.651756	0.981351	0.025*	
C4	0.8284 (3)	0.7197 (3)	0.87846 (17)	0.0225 (4)	
H4	0.798549	0.759683	0.948670	0.027*	
C8	0.2229 (3)	0.5089 (3)	0.92254 (17)	0.0237 (5)	
H8	0.168203	0.527811	0.988179	0.028*	
C2	1.0514 (3)	0.7057 (3)	0.74756 (18)	0.0241 (5)	
H2	1.176548	0.735707	0.727006	0.029*	
C16	0.6177 (3)	0.0264 (3)	0.83346 (19)	0.0266 (5)	
C1	0.9066 (3)	0.5979 (2)	0.67581 (17)	0.0216 (4)	
H1	0.934595	0.555208	0.605681	0.026*	
C3	1.0114 (3)	0.7691 (3)	0.84983 (18)	0.0254 (5)	
H3	1.107711	0.845362	0.899683	0.030*	

### Atomic displacement parameters (Å^2^)

**Table:**

	*U*^11^	*U*^22^	*U*^33^	*U*^12^	*U*^13^	*U*^23^
Cu1	0.02295 (16)	0.01815 (15)	0.01013 (14)	0.00641 (11)	0.00275 (10)	0.00076 (10)
Cl1	0.0282 (3)	0.0213 (2)	0.0128 (2)	0.0121 (2)	0.00591 (19)	0.00269 (19)
S1	0.0221 (3)	0.0223 (3)	0.0156 (3)	0.0060 (2)	−0.00080 (19)	−0.0002 (2)
F2	0.0382 (9)	0.0427 (9)	0.0461 (9)	0.0246 (7)	0.0131 (7)	0.0132 (7)
F1	0.0508 (10)	0.0437 (9)	0.0241 (7)	0.0128 (8)	−0.0061 (7)	0.0120 (7)
F3	0.0458 (10)	0.0206 (7)	0.0667 (12)	0.0001 (7)	−0.0117 (8)	0.0074 (7)
O1	0.0284 (8)	0.0199 (7)	0.0201 (8)	0.0056 (6)	0.0030 (6)	0.0021 (6)
O3	0.0230 (8)	0.0371 (10)	0.0371 (10)	0.0075 (7)	0.0075 (7)	0.0066 (8)
O2	0.0400 (10)	0.0429 (11)	0.0171 (8)	0.0111 (9)	−0.0078 (7)	−0.0016 (7)
N3	0.0262 (9)	0.0162 (8)	0.0132 (8)	0.0065 (7)	0.0020 (7)	0.0028 (7)
N2	0.0215 (9)	0.0152 (8)	0.0130 (8)	0.0063 (7)	0.0008 (6)	0.0021 (6)
N1	0.0239 (9)	0.0182 (8)	0.0133 (8)	0.0075 (7)	0.0024 (7)	0.0038 (7)
C11	0.0269 (11)	0.0159 (9)	0.0149 (9)	0.0073 (8)	0.0010 (8)	0.0037 (8)
C5	0.0248 (11)	0.0190 (10)	0.0141 (9)	0.0087 (8)	0.0032 (8)	0.0043 (8)
C10	0.0237 (10)	0.0167 (9)	0.0157 (10)	0.0073 (8)	0.0014 (8)	0.0039 (8)
C6	0.0234 (10)	0.0152 (9)	0.0144 (9)	0.0058 (8)	0.0014 (8)	0.0030 (8)
C9	0.0227 (11)	0.0225 (10)	0.0186 (10)	0.0053 (9)	0.0042 (8)	0.0039 (8)
C15	0.0332 (12)	0.0176 (10)	0.0144 (10)	0.0080 (9)	0.0016 (8)	0.0031 (8)
C14	0.0362 (12)	0.0149 (9)	0.0167 (10)	0.0056 (9)	−0.0045 (9)	0.0011 (8)
C12	0.0247 (11)	0.0182 (10)	0.0205 (10)	0.0065 (8)	0.0017 (8)	0.0054 (8)
C13	0.0259 (11)	0.0158 (10)	0.0246 (11)	0.0040 (8)	−0.0045 (9)	0.0039 (8)
C7	0.0256 (11)	0.0210 (10)	0.0122 (9)	0.0061 (9)	0.0010 (8)	0.0000 (8)
C4	0.0261 (11)	0.0241 (11)	0.0157 (10)	0.0088 (9)	0.0031 (8)	0.0020 (8)
C8	0.0282 (12)	0.0277 (11)	0.0134 (10)	0.0087 (9)	0.0055 (8)	0.0025 (8)
C2	0.0215 (11)	0.0285 (11)	0.0233 (11)	0.0074 (9)	0.0034 (8)	0.0093 (9)
C16	0.0308 (12)	0.0197 (10)	0.0240 (11)	0.0049 (9)	−0.0022 (9)	0.0010 (9)
C1	0.0266 (11)	0.0250 (11)	0.0166 (10)	0.0114 (9)	0.0055 (8)	0.0075 (8)
C3	0.0256 (11)	0.0275 (11)	0.0198 (11)	0.0063 (9)	−0.0014 (9)	0.0036 (9)

### Geometric parameters (Å, º)

**Table:**

Cu1—Cl1	2.2265 (5)	C10—C9	1.394 (3)
Cu1—Cl1^i^	2.7660 (6)	C6—C7	1.387 (3)
Cu1—N3	2.0278 (19)	C9—H9	0.9500
Cu1—N2	1.9420 (17)	C9—C8	1.390 (3)
Cu1—N1	2.0397 (18)	C15—H15	0.9500
S1—O1	1.4466 (17)	C15—C14	1.394 (3)
S1—O3	1.4409 (18)	C14—H14	0.9500
S1—O2	1.4392 (17)	C14—C13	1.376 (3)
S1—C16	1.826 (2)	C12—H12	0.9500
F2—C16	1.331 (3)	C12—C13	1.401 (3)
F1—C16	1.335 (3)	C13—H13	0.9500
F3—C16	1.337 (3)	C7—H7	0.9500
N3—C11	1.362 (3)	C7—C8	1.392 (3)
N3—C15	1.339 (3)	C4—H4	0.9500
N2—C10	1.335 (3)	C4—C3	1.391 (3)
N2—C6	1.336 (3)	C8—H8	0.9500
N1—C5	1.364 (3)	C2—H2	0.9500
N1—C1	1.336 (3)	C2—C1	1.384 (3)
C11—C10	1.481 (3)	C2—C3	1.386 (3)
C11—C12	1.380 (3)	C1—H1	0.9500
C5—C6	1.479 (3)	C3—H3	0.9500
C5—C4	1.388 (3)		
			
Cl1—Cu1—Cl1^i^	89.944 (18)	C8—C9—C10	117.9 (2)
N3—Cu1—Cl1^i^	90.30 (5)	C8—C9—H9	121.0
N3—Cu1—Cl1	99.82 (5)	N3—C15—H15	118.9
N3—Cu1—N1	159.58 (7)	N3—C15—C14	122.2 (2)
N2—Cu1—Cl1^i^	90.83 (5)	C14—C15—H15	118.9
N2—Cu1—Cl1	179.20 (5)	C15—C14—H14	120.4
N2—Cu1—N3	80.39 (7)	C13—C14—C15	119.1 (2)
N2—Cu1—N1	80.11 (7)	C13—C14—H14	120.4
N1—Cu1—Cl1	99.60 (5)	C11—C12—H12	120.7
N1—Cu1—Cl1^i^	95.97 (5)	C11—C12—C13	118.7 (2)
Cu1—Cl1—Cu1^i^	90.056 (18)	C13—C12—H12	120.7
O1—S1—C16	102.05 (10)	C14—C13—C12	119.2 (2)
O3—S1—O1	114.97 (10)	C14—C13—H13	120.4
O3—S1—C16	103.57 (11)	C12—C13—H13	120.4
O2—S1—O1	114.19 (11)	C6—C7—H7	121.0
O2—S1—O3	115.73 (12)	C6—C7—C8	118.1 (2)
O2—S1—C16	103.90 (11)	C8—C7—H7	121.0
C11—N3—Cu1	113.61 (14)	C5—C4—H4	120.6
C15—N3—Cu1	127.30 (15)	C5—C4—C3	118.8 (2)
C15—N3—C11	118.61 (19)	C3—C4—H4	120.6
C10—N2—Cu1	118.38 (14)	C9—C8—C7	121.0 (2)
C10—N2—C6	123.04 (18)	C9—C8—H8	119.5
C6—N2—Cu1	118.58 (14)	C7—C8—H8	119.5
C5—N1—Cu1	113.57 (14)	C1—C2—H2	120.5
C1—N1—Cu1	127.51 (15)	C1—C2—C3	119.1 (2)
C1—N1—C5	118.60 (19)	C3—C2—H2	120.5
N3—C11—C10	114.06 (19)	F2—C16—S1	111.78 (16)
N3—C11—C12	122.2 (2)	F2—C16—F1	107.3 (2)
C12—C11—C10	123.8 (2)	F2—C16—F3	107.8 (2)
N1—C5—C6	114.01 (18)	F1—C16—S1	110.99 (16)
N1—C5—C4	121.9 (2)	F1—C16—F3	106.5 (2)
C4—C5—C6	124.13 (19)	F3—C16—S1	112.25 (17)
N2—C10—C11	113.09 (18)	N1—C1—C2	122.5 (2)
N2—C10—C9	119.91 (19)	N1—C1—H1	118.7
C9—C10—C11	127.0 (2)	C2—C1—H1	118.7
N2—C6—C5	113.29 (18)	C4—C3—H3	120.5
N2—C6—C7	120.03 (19)	C2—C3—C4	119.1 (2)
C7—C6—C5	126.68 (19)	C2—C3—H3	120.5
C10—C9—H9	121.0		
			
Cu1—N3—C11—C10	−7.7 (2)	N1—C5—C4—C3	0.2 (3)
Cu1—N3—C11—C12	170.55 (16)	C11—N3—C15—C14	1.5 (3)
Cu1—N3—C15—C14	−170.07 (15)	C11—C10—C9—C8	−178.6 (2)
Cu1—N2—C10—C11	−0.7 (2)	C11—C12—C13—C14	0.8 (3)
Cu1—N2—C10—C9	179.33 (15)	C5—N1—C1—C2	−1.1 (3)
Cu1—N2—C6—C5	−1.4 (2)	C5—C6—C7—C8	−177.9 (2)
Cu1—N2—C6—C7	179.33 (15)	C5—C4—C3—C2	−1.7 (3)
Cu1—N1—C5—C6	7.0 (2)	C10—N2—C6—C5	179.27 (18)
Cu1—N1—C5—C4	−172.78 (16)	C10—N2—C6—C7	0.0 (3)
Cu1—N1—C1—C2	171.97 (16)	C10—C11—C12—C13	178.98 (19)
O1—S1—C16—F2	−60.24 (18)	C10—C9—C8—C7	−0.1 (3)
O1—S1—C16—F1	59.49 (19)	C6—N2—C10—C11	178.63 (18)
O1—S1—C16—F3	178.50 (17)	C6—N2—C10—C9	−1.4 (3)
O3—S1—C16—F2	−179.95 (16)	C6—C5—C4—C3	−179.6 (2)
O3—S1—C16—F1	−60.22 (19)	C6—C7—C8—C9	−1.1 (3)
O3—S1—C16—F3	58.8 (2)	C15—N3—C11—C10	179.69 (18)
O2—S1—C16—F2	58.73 (19)	C15—N3—C11—C12	−2.1 (3)
O2—S1—C16—F1	178.47 (17)	C15—C14—C13—C12	−1.5 (3)
O2—S1—C16—F3	−62.5 (2)	C12—C11—C10—N2	−172.60 (19)
N3—C11—C10—N2	5.6 (3)	C12—C11—C10—C9	7.4 (3)
N3—C11—C10—C9	−174.4 (2)	C4—C5—C6—N2	175.9 (2)
N3—C11—C12—C13	1.0 (3)	C4—C5—C6—C7	−4.9 (3)
N3—C15—C14—C13	0.3 (3)	C1—N1—C5—C6	−178.99 (18)
N2—C10—C9—C8	1.4 (3)	C1—N1—C5—C4	1.3 (3)
N2—C6—C7—C8	1.2 (3)	C1—C2—C3—C4	1.9 (3)
N1—C5—C6—N2	−3.8 (3)	C3—C2—C1—N1	−0.4 (3)
N1—C5—C6—C7	175.3 (2)		

Symmetry code: (i) −*x*+1, −*y*+1, −*z*+1.
